# Structural Characters and Pharmacological Activity of Protopanaxadiol-Type Saponins and Protopanaxatriol-Type Saponins from Ginseng

**DOI:** 10.1155/2024/9096774

**Published:** 2024-06-24

**Authors:** Lancao Zhang, Xiang Gao, Chunhui Yang, Zuguo Liang, Dongsong Guan, Tongyi Yuan, Wenxiu Qi, Daqing Zhao, Xiangyan Li, Haisi Dong, He Zhang

**Affiliations:** ^1^ Northeast Asia Research Institute of Traditional Chinese Medicine Changchun University of Chinese Medicine, Changchun 130117, China; ^2^ College of Pharmacy Changchun University of Chinese Medicine, Changchun 130117, China; ^3^ Tuina Department The Third Affiliated Hospital to Changchun University of Traditional Chinese Medicine, Changchun 130117, China; ^4^ Quality Testing Laboratory, Haerbin Customs District 150008, Foshan, China; ^5^ Research Center of Traditional Chinese Medicine The Affiliated Hospital to Changchun University of Chinese Medicine, Changchun 130021, China

## Abstract

Ginseng has a long history of drug application in China, which can treat various diseases and achieve significant efficacy. Ginsenosides have always been deemed important ingredients for pharmacological activities. Based on the structural characteristics of steroidal saponins, ginsenosides are mainly divided into protopanaxadiol-type saponins (PDS, mainly including Rb1, Rb2, Rd, Rc, Rh2, CK, and PPD) and protopanaxatriol-type saponins (PTS, mainly including Re, R1, Rg1, Rh1, Rf, and PPT). The structure differences between PDS and PTS result in the differences of pharmacological activities. This paper provides an overview of PDS and PTS, mainly focusing on their chemical profile, pharmacokinetics, hydrolytic metabolism, and pharmacological activities including antioxidant, antifatigue, antiaging, immunodulation, antitumor, cardiovascular protection, neuroprotection, and antidiabetes. It is intended to contribute to an in-depth study of the relationship between PDS and PTS.

## 1. Introduction

Over the centuries, traditional Chinese medicines have been widely used to treat numerous diseases for their perceived effectiveness, fewer side effects, and relatively low cost, which are currently attracting the most attention from researchers worldwide as alternative and supplemental medicines [1]. Ginseng is a traditional Chinese medicine, also known as the God of grass, the king of herbs, that is the dried root or rhizome of the Araliaceae plant ginseng (*Panax ginseng* C.A. Meyer). Panax is derived from the word “panacea,” which means a cure for all diseases and a source of longevity as well as physical strength and resistance [2].

Ginseng includes diverse chemical constituents, such as ginsenosides, fatty acids, polysaccharides, and polyacetylenes. The extensive pharmacological activities can be attributed primarily to the triterpene saponins [3]. Saponins include four-ring triterpene saponins (protopanaxadiol-type saponin (PDS) and protopanaxatriol-type saponin (PTS)) and five-ring triterpene saponins (oleanane-type saponin). PDS and PTS are major effective components of ginseng. Modern pharmacological studies have proved that PDS and PTS have various effects such as antifatigue, antishock, antitumor, blood sugar regulation, immunoregulation, and the protection of liver, kidney, heart, spleen, thymus, and so on. However, PDS and PTS are poorly intestinal absorption and have low oral bioavailability because of their unfavorable physicochemical traits, such as large molecular mass (>500 Da), high hydrogen-bonding capacity (>12), high molecular flexibility (>10), and biliary excretion [4]. Several ginsenosides such as Rb1, Rb2, Rc, and Rd are absorbed into the bile and show remarkably long elimination half-life (20∼40 h in mice) [5]. Some ginsenosides with large molecular mass (Ra1, Re, Rb1, Rb2, etc.) are hydrolyzed by intestinal bacteria to produce secondary saponins including rare ginsenosides compound K (CK), Rh1, Rh2, Rg3, and Rg5, the later exert the better bioactivity and higher bioavailability. The protopanaxadiol (PPD)-type ginsenoside Rb1 is hydrolyzed into Rd ⟶ F2 ⟶ CK ⟶ 20(*S*)-PPD [6]; the protopanaxatriol (PPT)-type ginsenoside Rg1 is hydrolyzed into Rh1 ⟶ 20(*S*)-PPT [7]. The human study showed that CK and Rh1 might reach the systemic circulation [8], and the plasma protein-binding ratio of Rd and CK was 80∼95% and 99% in rats, respectively [4]. The diversities of PDS and PTS in the structural forms show a close relationship between biological and pharmacological actions. Therefore, the studies on the structure and transformation of PDS and PTS are important in understanding their biological effects and the possible health benefits of ginsenosides in humans.

## 2. Chemical Structure Characteristics of PDS and PTS

**Figure 1:**
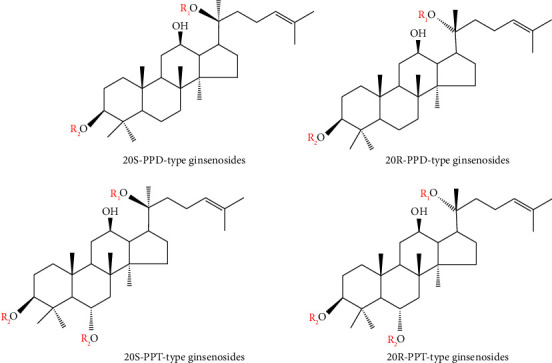
The structures of PPD-type ginsenosides core and PPT-type ginsenosides core.

Ginsenosides comprise a hydrophobic four-ring steroidal system and a diverse range of sugar, such as glucose (glc), rhamnose (rha), xylose (xyl), and arabinose (ara) attached to the C-3, C-6, and C-20 positions. Most ginsenosides belong to the dammarane type, which divide into four types, i.e., PDS contains a hydrogen atom at C-6 (Rb1, Rb2, Rb3, Rc, Rd, Rg3, Rh2, etc.), the PTS group contains a C-6 sugar side-chain (Re, Rf, Rg1, Rg2, etc.), the oleanane type (Ro), and the ocotillol group (F11).  Figure 1 shows the chemical structures of the two basic ginseng saponin types, and Table 1 summarizes the chemical structure characteristics of the saponin side chain in different ginsenosides. The main ginsenosides include the PPD-type ginsenoside Rb1, Rb2, Rc, Rd, and PPT-type ginsenoside Re and Rg1 in the ginseng, which account for more than 90% of the total ginsenoside content.

## 3. Pharmacokinetics of PDS and PTS

The chemical structures of PDS and PTS are tetracyclic triterpene saponins consisting of different positions and numbers of hydroxyl groups; therefore, the absorption, distribution, elimination metabolism, and bioavailability of PDS and PTS are obvious differences in the body. At a given dose, the *T*_max_ of oral PPD and PPT was 1.82 h and 0.58 h, respectively, and the absorption of PPT was faster than that of PPD. Meanwhile, PPT was eliminated rapidly from the body with an average *T*_1/2_ for 0.80 h and *Cl* for 4.27 L/h/kg after i.v. administration; however, PPD was eliminated relatively slowly with a *T*_1/2_ for 6.25 h and *Cl* for 0.98 L/h/kg. The *C*_max_ was 0.13 *μ*g/mL and 1.04 *μ*g/mL for PPT and PPD. The bioavailability of PPT and PPD was 3.69% and 48.12%, respectively. The absorption and bioavailability of PPD were higher than those of PPT, and the elimination rate of PPD was slower than that of PPT [16]. The *T*_max_ of oral Rb1, Rb2, and Rb3 was 2 h, 4.8 h, and 1.5 h, respectively; the AUC_0-36h_ of Rb1 (63.5 mg/L·h) was about 10 times larger than that of Rb2 (6.4 mg/L·h) and was 1.7 times larger than that of Rb3 (37.4 mg/L·h). *T*_max_ showed that the absorption of Rb3 was relatively fast, and Rb1 was relatively slow; AUC_0-36h_ showed that the absorption of Rb1 was more than Rb2 and Rb3. The *T*_1/2_ of Rb1, Rb2 and Rb3 was 12.5 h, 15.4 h, and 24.9 h through the tail vein, respectively; they were slowly eliminated *in vivo*, the elimination of Rb1 was the fastest, while Rb3 was the slowest among Rb1, Rb2, and Rb3. The bioavailability of Rb1, Rb2, and Rb3 was 0.78%, 0.08, and 0.52%, respectively; they were poor oral absorption [17]. The AUC_0-*t*_ of Rb1 and Rd (36.64 mg/L·h and 20.68 mg/L·h) were higher than those of Re and Rg1 (3.01 mg/L·h and 13.89 mg/L·h). The *T*_1/2_ of Rb1 and Rd (20.15 h and 18.15 h) were longer than those of Re and Rg1 (1.01 h and 5.01 h). The MRT_0-t_ (mean retention time) of Rb1 and Rd (19.09 h and 19.09 h) was more than those of Re and Rg1 (2.44 h and 3.16 h) [18]. The *T*_1/2_ of Rb1, Rd, Re, and Rg1 were 19.7 h, 48.9 h, 0.2 h, and 0.2 h after intravenous Shengmai injection in rats, respectively [19]. *T*_max_ of Rb1, Rd, Rg3, CK, and PPD were 2.0 h, 2.8 h, 2.6 h, 6.0 h, and 7.2 h in mice via oral red ginseng extract, respectively [5]. *T*_1/2_ of Re, Rg2, Rg1, Rh1, and PPT were 0.2 h, 0.1 h, 0.1 h, 0.4–0.7 h, and 1.9 h, respectively [20]. The *Cl* of Rb1, Rd, Re, and Rg1 was 0.08 L/h/kg, 0.01 L/h/kg, 1.8 L/h/kg, and 1.1 L/h/kg, respectively [19]. Although the ginsenosides were poorly absorbed orally, the absorption of PDS was significantly higher than PTS *in vivo*. The *T*_1/2_ of PDS was higher than that of PTS, and the *Cl* of PDS was slower than that of PTS. PDS is trihydroxy substituted at C-3, C-12, and C-20, while PTS is at C-3, C-6, C-12, and C-20, such an elimination rate of PTS was faster than that of PDS because of the extra C-6 hydroxyl group [21]. Meanwhile, the *T*_max_ was increased with a reduction in the number of glycosides in the PDS.

After absorbing, ginsenosides are widely distributed in various organs of the body. 9, 12, 12, 12, and 14 ginsenosides were detected in the brain (Rb1 > Rd > Rb2 > Rc > Re > Rb3 > Rg1 > Rf > nR1), heart (Rb1 > Rd > Rb2 > Rc > Rb3 > Re > Rg1 > Rg3 > Rf > Ro > nR1 > Rg2), spleen (Rd > Rb1 > Rb2 > Rc > Ro > Rb3 > Re > Rg1 > Rg3 > Rf > nR1 > Rg2), liver (Rd > Rb1 > Rb2 > Rg3 > Rc > Re > Rg1 > Rb3 > Rf > Ro > Rg2 > nR1), and kidney (Rd > Rb1 > Rb2 > Re > Rc > Rg1 > Rb3 > Rg3 > Rf > nR1 > CK > Rg2 > Rh1 > Ro) by a single intravenous bolus dose of Shenmai injection (SMI) [19]. The ginsenoside content of the kidney (8.33∼6250 ng/g) was highest, followed by the spleen (1.87∼2117 ng/g), heart (0.31∼1785 ng/g), liver (0.09∼1297 ng/g), and brain (0.16∼132 ng/g) [19, 20]. The systemic exposure of PDS was significantly greater than that of PTS after oral administration of ginsenosides. However, systemic exposure has obvious differences between PPD and PPT that needs widespread attention in clinical for optimizing dosage regimens and predicting outcomes in patients receiving ginseng-based therapy.

## 4. Hydrolytic Metabolism

**Figure 2:**
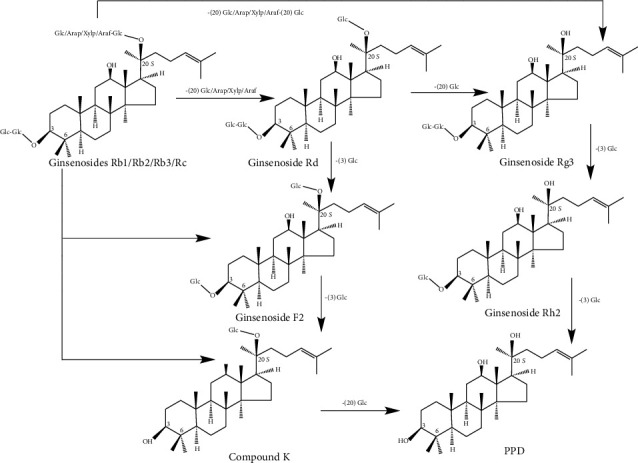
Schematic diagram of PDS *in vivo* metabolism.

**Figure 3:**
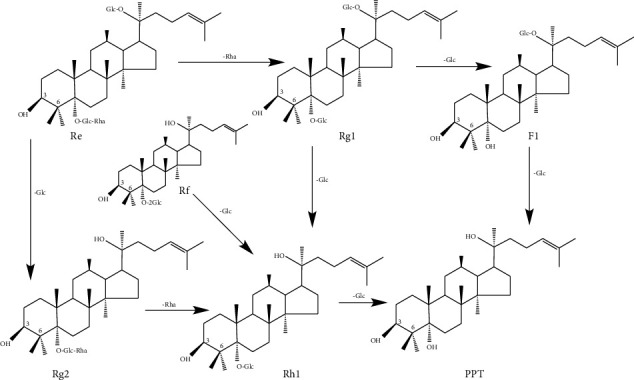
Schematic diagram of PTS *in vivo* metabolism.

Most ginsenosides of PDS and PTS are deglycosylated in the gastrointestinal tract via the action of intestinal bacteria, acid, alkali, and/or enzyme. Reports suggested that the hydrophilic ginsenosides (nR1, Rb1, Rg1, Rc, Re, and R1) were deglycosylated into hydrophobic secondary ginsenosides or aglycones (Rd, Rh2, CK, PPT, and PPD) by Lactobacillus plantarum-mediated fermentation [22, 23]. *β*-glucosidases were the important enzymes in the biological transformation process of ginsenosides, which could hydrolyze Rb1/Rb2/Rb3/Rc to generate the deglycosylation minor ginsenosides Rd, F2, CK, Rg3, Rh2, and PPD in the intestinal bacteria (Figure 2) [24, 25]. *β*-glucosidases also hydrolyzed Re to generate the minor ginsenosides Rg1, Rg2, F1, Rh1, and PPT (Figure 3) [26]. All the metabolites are nonpolar compared to the parental components, which are easier to absorb to cell membranes and exhibit stronger bioavailability; these properties further determine their pharmacological efficacies. Meanwhile, some researchers found that several intestinal bacteria were essential to hydrolyze ginsenosides, such as *Lactobacillus*, *Bacteroides*, *Bifidobacterium*, *Eubacterium*, *Prevotella*, and *Streptococcus* [27, 28]. Ginseng polysaccharides enhance the systemic exposure, metabolism, and intestinal absorption of ginsenosides by affecting gut microbial [29, 30].

## 5. Pharmacological Effects and Mechanisms of PDS and PTS

### 5.1. General Effects of Ginseng Saponins

#### 5.1.1. AntiOxidant

Oxidative stress (OS) is a state of imbalance between oxidative and antioxidant effects in the body that tends to oxidize, leads to inflammatory infiltration of neutrophils, secretion of proteases, and the production of large amounts of oxidative intermediates, especially reactive oxygen species (ROS), and which damages the normal organs and leads to a progressive loss of vital physiological functions. Most of the ginsenosides, including total ginsenosides, and monomer ginsenosides, such as Rb1, Rg1, Re, Rd, CK, and Rh1, showed the antioxidative effect *in vivo* and *in vitro*. PDS acted as an antioxidant to protect erythrocytes against 2,2′-azobis (2-amidinopropane hydrochloride) (AAPH)-induced hemolysis; however, PTS showed cytotoxicity to promote the hemolysis [31]. The antioxidative activity of Rg2 (IC50 value 14.12) was better than Rb1 (IC50 value 9.67) in AAPH-induced hemolysis; meanwhile, the antioxidation of the *Z*-configuration was greater than the *E*-configuration of pseudo-Rg2, pseudo-Rh2, pseudo-Rg3, and pseudo-Rh1 [32]. The orders of antioxidative effect were Rc > Rb1 and Re > Rd > R1 > Rg1 > Rb3 > Rh1 [31]. These results showed that the antioxidative effect of PDS was stronger than that of PTS, most of the *Z* configuration was better than the *E* configuration, and the antioxidative activity of ginsenosides with low-sugar chain was higher than ginsenosides with high-sugar chain in PDS. Rb1, Rb2, Re, and Rg1 mainly reduced astrocytic death and ROS formation by increasing the activity of superoxide dismutase (SOD), glutathione peroxidases (GPx), and glutathione reductase (GR) [33]. Rg3 significantly inhibited oxidative stress in cyclophosphamide-induced mice and exerted antioxidant effects by increasing the activities of catalase (CAT), SOD, and lysozyme and decreasing levels of xanthine oxidase and malondialdehyde (MDA) [34]. Rh2 inhibited the oxidative stress of porcine oocytes induced by H_2_O_2_ by regulating SIRT1 expression and mitochondrial activity [35]. CK protected human retinal pigment epithelial (RPE) cells from oxidative stress-stimulated damage by activating the Nrf2/HO-1 signaling [36]. PTS well improved DNA damage and excessive activation of the DNA repair enzyme PARP-1 and inhibited depletion of the intracellular substrate NAD+ of endothelial cells induced by H2O2, which also reversed the decrease of the ATP/ADP ratio and the GSH/GSSG ratio by increasing the activities of GR and glutathione peroxidase (GSH-Px) [37]. Rg1 exerted the antioxidant effect by activating the Akt/GSK3*β*/NRF2 pathway and reducing the cell apoptosis in the D-gal-induced model mice [38]. Rh4 increased the levels of SOD, glutathione (GSH), and nitric oxide (NO) and decreased the levels of MDA, tumor necrosis factor-*α* (TNF-*α*), interleukin 6 (IL-6), and interleukin1*β* (IL-1*β*) in ethanol-induced gastric ulcer model rat, which inhibited the inflammation and oxidative stress by blocking the MAPK/NF-*κ*B pathway [39]. These results show that the antioxidative effect of PDS is stronger than PTS *in vivo* and *in vitro*.

#### 5.1.2. Antifatigue

Healthy and sick humans frequently feel fatigued under physiological conditions. Some studies have shown that most ginsenosides improve fatigue syndrome. Wang et al. reported that PDS significantly increased the lactic dehydrogenase (LDH) and muscle glycogen content and decreased the blood lactate; whereas, PTS mainly increased the liver glycogen content of mice in forced swimming test (FST). PDS could reduce the lipid peroxide (LPO) content in liver, kidney, heart, and skeletal muscle tissues, decrease plasma MDA levels, improve the SOD activity in lung tissue and erythrocyte, and have a recovery effect on exercise fatigue and sport injuries caused by imbalance of oxidative and antioxidant systems. Rb1 obviously decreased the ROS and malondialdehyde release and elevated the activity of SOD of skeletal muscle through the PI3K/Akt/Nrf2 pathway in aged rats [40]. Rg3 increased the serum levels of total cholesterol (TC), serum triglyceride (TG), and LDH and elevated the activity of SOD of skeletal muscles by activating SIRT1 and inhibiting the p53 transcription factor [41]. Meanwhile, 20(R)-Rg3 entrapped in chitosan microspheres exerted antifatigue effects by increasing the residence time of Rg3 and promoting its absorption by nasal mucosa [42]. Rg1 exerted the antifatigue effect by impacting the metabolism of taurine and mannose 6-phosphate by epidermal growth factor receptor (EGFR) [43]. Some ginsenosides targeted the creatine kinase muscle type (CKMM) in skeletal muscle [44]. PPD and PPT were the final metabolites of PDS and PTS. Cheng et al. reported that PPD was the best activator of CKMM among the 12 dammarane-type compounds, and exerted an antifatigue effect by increasing the level of tissue phosphocreatine and delaying exercise-induced lactate accumulation. The cyclization of the side chain, the hydroxyl group of C^6^, and the glycosylation of C-3, C-6, and C-20 in PDS and PTS reduced the activation effect of ginsenosides on the CKMM compared to PPD and PPT [44, 45]. These results show that PDS and PTS have antifatigue effects. In some results, the antifatigue effect of PDS is stronger than that of PTS, and the antioxidant activity of ginsenosides with low-sugar chain was higher than ginsenosides with high-sugar chain in PDS and PTS.

#### 5.1.3. Antiaging

Aging is mainly characterized by a progressive dysfunction of metabolism and various physiological roles. PDS and PTS are important antiaging drugs that could promote the metabolism and proliferation of stem cells, protect the skin, brain, heart, and nerves, enhance mitochondrial function and telomerase activity, and maintain the intestinal flora [46]. Xu et al. reported that Rb1 and Rg1 reduced ROS production, increased CAT activity, elevated the mtDNA content and ATP level, and attenuated the MMP depolarization of primary mouse astrocytes injury induced by oxygen-glucose deprivation/reoxygenation (OGD/R). The protective effect of Rb1 on OGD/R-induced injury was stronger than that of Rg1 [47]. Rb1 attenuated metabolic disorders of the aging-induced mouse by regulating the cell cycle and apoptotic pathway of cardiomyocytes [48]. Rg1 exerted an antiaging function on hematopoietic stem cells by activating the SIRT3/SOD2 pathway, the p16(INK4a)-Rb and p19(Arf)-p53-p21(Cip/Waf1) signaling pathways [49], increasing the lengths and activity of the telomere, and restraining mitochondrial pathway-mediated apoptosis in aging rat induced by D-gal [50]. Also, Rg1 attenuated the cognitive capacity, senescence-related markers, and hippocampal neurogenesis and inhibited the expression of cellular senescence-associated genes p53, p21Cip1/Waf1, and p19Arf of the hippocampus in D-gal-induced aging rat [51]. Rh4 could target SIRT1 to inhibit oxidative stress and inflammation of aging skeletal muscle cells by activating the PGC-1*α*-TFAM and HIF-1*α*-c-Myc pathways and enhancing mitochondrial homeostasis [52]. Rg1, Re, and Rb1 were the major ginsenoside monomers that prolonged lifespan by activating NRF2/SKN-1, SIRT1/SIR 2.1, and FOXO/DAF-16 signaling pathways [53]. Ginsenosides could also exert antiaging effects by delaying the occurrence of inflammation. The transformed ginsenosides Rd, GypXVII, Rg2, and PPT significantly inhibited TNF-*α* and IL-6 production induced by LPS compared to Rb1, Re, and Rg1; meanwhile, the anti-inflammatory effect of Rd was stronger than that of PPT, Re, and Rg1 [54]. Rb1 inhibited the extracellular matrix degradation, matrix metalloproteinase (MMP) production, inflammatory cell infiltration, and vascular smooth muscle cell (VSMC) dysfunction induced by Ang II through inhibiting the JNK and p38 signaling pathways; however, Rg1 could not improve the above indices [55]. Zhang et al. reported that CK on acute arthritis showed the best anti-inflammatory effect among Rg1, Rg3, Rg5, Rb1, Rh2, and CK [56]. These results show that PDS on the antiaging effect is stronger than that of PTS.

### 5.2. Immunomodulatory Effect

**Figure 4:**
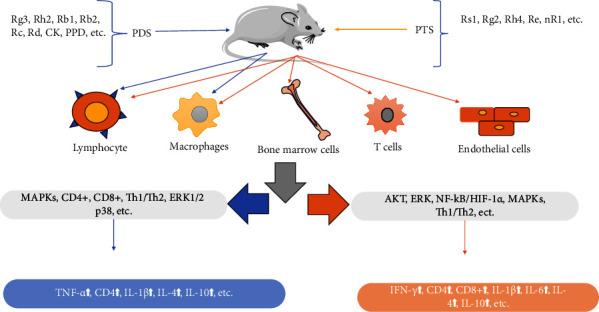
The immunostimulatory role of ginsenosides *in vivo*.

**Figure 5:**
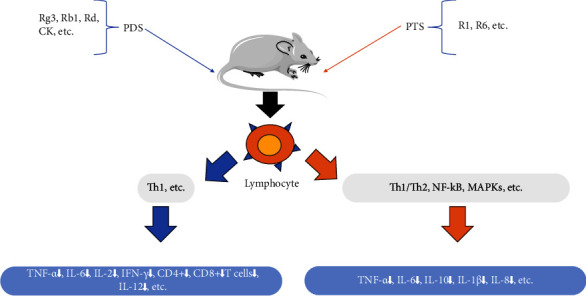
The immunosuppressive role of ginsenosides *in vivo*.

The immune system is our main defense barrier against the invasion of pathogens. It detects and removes foreign substances and kills pathogens and other microorganisms. PDS (including Rg3, Rh2, Rb1, Rb2, Rc, Rd, CK, and PPD) and PTS (including Rg1, Rg2, Rh4, Re, and nR1) could activate the immune responses in some diseases, such as lung injury, liver injury, and asthma (Figure 4); however, PDS (including Rg3, Rb1, Rd, and CK) and PTS (R1 and R6) showed immunosuppressive properties in some diseases, such as rheumatoid arthritis (RA), septic shock, and sepsis (Figure 5) [57]. Zhang et al. reported that both PDS and PTS protected the immunomodulatory of bone marrow, thymus, and spleen in immunosuppressed mice induced by cyclophosphamide (CP), they improved the hematopoiesis-related cytokines and inflammatory cytokines, promoted the cell cycle and inhibited the apoptosis of bone marrow, and increased the CD4^+^ cell of spleen. However, the prevention effect of PDS was stronger than PTS in some parameters, including red blood cells, hemoglobin, IL-1*β*, IL-4, IL-10, TNF-*α*, CD4^+^, and thymus index [58]. CP is a chemotherapy drug that causes myelosuppression and immunosuppression, which disrupts the balance between Th1 and Th2. PDS and PTS promoted lymphocyte, macrophage, and endothelial cells to release cytokines, which regulated the immune response and the dynamic balance between Th1 and Th2. Rb1 regulated the immune function by enhancing the T-lymphocytes subsets (CD4^+^ and CD8^+^), the expressions of cytokines (IL-2, IL-6, IFN-*γ*, and TNF-*α*), and the production of immunoglobulins (IgA, IgM, and IgG) of immune injury mice induced by deoxynivalenol [59]. Rb2 bound to target proteins of immune regulation to improve the pathological characteristics of immunosuppression mice induced by CP, which enhanced the viability of natural killer cells and boosted the expression of IFN-*γ*, TNF-*α*, IL-2, and IgG [60]. CK inhibited the macrophage phagocytosis by decreasing overexpression of *β*-arrestin2, G*α*i, TLR4, and NF-*κ*B in the collagen-induced arthritis (CIA) mouse, which reduces the proportion of M1 by inhibiting the colocalization of *β*-arrestin2 with G*α*i and TLR4 with G*α*i and promoting TLR4 coupling with Gas [61]; meanwhile, CK downregulated dendritic cells (DCs) priming of T-cell activation and suppressed migration and of signaling CCL21/CCR7-mediated T cells and DCs in mouse with collagen-induced arthritis (CIA) [62]. Rg1 regulated the innate immune response in macrophages by differentially modulating the expression of related cytokine and the NF-*κ*B and PI3K/Akt/mTOR pathways [63]. Rg1 attenuates IL-1*β*-induced inflammation and apoptosis of podocytes by increasing the NRF2 pathway [64]. Re inhibited the production of IFN-*γ* and immunity-related GTPase family M (IRGM) in CD4+ T cell; however, they had no changes in other autophagy-related signaling molecules (including ERK, p38, and AKT-mTOR-p70S6k) [65]. Minor ginsenosides Rg2 and Rh1 reduced LPS-induced PKC*δ* expression and translocation to the membrane, resulting in p38-STAT1 activation and NF-*κ*B translocation; especially, the combined Rg2 and Rh1 further enhanced the blocking effect and reduced the levels of TNF-*α*, IL-1*β*, and IFN-*β* [66]. PDS and PTS have good immune regulation by regulating ARK, MAPK, and ERK1/2 signaling pathways and balance between Th1 and Th2; PDS showed strong immune regulatory effects compared to PTS in some parameters.

### 5.3. Anticancer

Some ginsenosides have been widely believed to effectively inhibit the proliferation, migration, and invasion of various tumor cells and promote cell apoptosis; meanwhile, they also relieve immune suppression and myelosuppression caused by chemotherapy drugs. Due to the dual therapeutic effects, ginsenosides are increasing concern and attention. PDS and PTS were important anticancer ingredients, and the anticancer effect of PDS was stronger than that of PTS. The polarity of ginsenosides influenced cellular uptake. Lee et al. thought that PTS with the C-6 OH group had high polarity compared to PDS; the ginsenosides with low polar possessed higher cytotoxic activity to cancer cells; such PDS exhibited more cytotoxic and efficient cellular uptake on MCF-7 and MDA-MB231 cells compared with PTS [67]. Meanwhile, Dong et al. found that the cytotoxic potency of the hydrolysates of PDS and PTS was stronger than the original ginsenosides on LLC1 and CCD19Lu cells [68], and the presence of sugars in PPD and PPT aglycone structures reduced the toxicity on the human leukemia cells (THP-1) [69].

**Figure 6:**
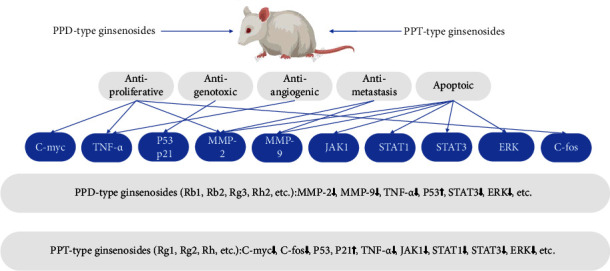
Potential anticancer-signaling pathways of PPD and PPT.

The anticancer effects of PPD, CK, Rh2, Rb1, and Rb2 in PDS are popular research, and Rg1 and PPT are also studied (Figure 6). At present, PPD shows pleiotropic anticancer capabilities in most cancer cell lines [70], which activated caspase-3, -7, -8, and -9 to induce rapid apoptosis and autophagy of human glioma cell SF188 and U87MG through caspase-dependent and -independent mechanisms, respectively [71]. PPD initiated the intrinsic and extrinsic apoptotic pathway of HepG2 cells by activating DR5, caspase-3, -8, -9, promoting the cleavage of PARP, and inhibiting the expression of Bcl-2 and Bcl-xl proteins. Zhu et al. found that ER was the molecular target of PPD, and PPD induces cancer cell apoptosis through the ER stress pathway [72]. CK, the important metabolite of PDS in the body, was almost nonexistent in ginseng. CK induced the cancer cell apoptosis by multiple signal pathways. The Akt/mTOR/c-Myc signaling pathway is involved in aerobic glycolysis and tumor growth. Shin et al. reported that CK induced apoptosis by inhibiting the expression of pro-PARP and procaspase 3 and blocking the Akt/mTOR/c-Myc pathway and the downstream proteins (HK2 and PKM2) of HepG2 and HCCs. CK also induced ER stress-related apoptosis of HL-60, HepG2, and SMMC-7721 cells by activating caspases-12, PERK, and IRE1 pathways and inhibiting STAT3 [73, 74]. TNF-related apoptosis-inducting ligand (TRAIL) is a potent cytokine that induces the cancer cell apoptosis. Chen et al. reported that CK enhanced the TRAIL-induced apoptosis in HCT116 colon cancer cells through autophagy-dependent and -independent (p53-CHOP pathway) DR5 upregulation [75]. Meanwhile, CK induced autophagy and apoptosis of HCC cells through ROS generation and JNK activation [76]. Kim et al. reported that CK increased the expression of p15 to induce the G1 phase arrest of HCT-116 cells by activating ATM/p53-p21 and FoxO3a-p27/p15 pathways [77]. Rg3 targets cancer stem cells and tumor angiogenesis to inhibit the growth and stemness of colorectal cancer progression [78]. Rh2 had a significant anticancer effect by inducing apoptosis, autophagy, and cell cycle arrest, inhibiting proliferation, invasion, metastasis, and angiogenesis, and reversing the drug resistance through regulating multiple signal pathways, such as the ER stress pathway, MAPK/JNK pathway, Src/ERK pathway, and PI3K/Akt/mTOR pathway [79–81]. Rg1 inhibited the proliferation, migration, and invasion of cancer cells through upregulating connexin 31 [82] and inhibiting TGF-*β*1-induced EMT [83], NF-*κ*B-mediated regulation of MMP-9 expression [84] in thyroid cancer, HepG2, and MCF-7 cells, respectively. PPT reduced the expression of tyrosinase-related protein-1 and -2 to inhibit the melanin synthesis and dendrite elongation of B16 melanoma cells through the CREB-MITF-tyrosinase signaling pathway [85]. Most ginsenosides in PDS showed strong anticancer effects; however, Rg1 and PPT in PTS had anticancer effects.

### 5.4. Effect on the Cardiovascular System

Cardiovascular disease is the main cause of morbidity and mortality in the world, and affects tens of millions of people every year. Atherosclerosis (AS) is the main foundation of pathology in heart attacks, strokes, and cerebral infarction. AS begins with inflammation and thrombosis, which are considered to be caused by the damage of the vascular endothelial cells (ECs) and the deposition of lipids [86]. In addition, hypercholesterolemia, hypertension, and diabetes can induce EC dysfunction to develop atherosclerosis. Over time, some inflammation factors and proatherogenic transcriptional factors promote the formation of atherosclerotic lesions, which cause vascular remodeling, occlusion of the vascular lumen, thrombus, and bleeding, subsequently leading to acute myocardial infractions or stroke or acute ischemia of any nearby organ [87]. The underlying mechanism of ginsenoside treatment of atherosclerosis is the inhibition of key steps in the development of the pathology, such as vascular smooth muscle cell (VSMC) proliferation, endothelial dysfunction, lipid deposition, oxidative stress, hyperlipidemia, chronic inflammation, and macrophage polarization [88]. We summarized the effect of PDS and PTS on the cardiovascular system from the following two areas: antiplatelet aggregation effects and abnormal vascular remodeling.

#### 5.4.1. Antiplatelet Aggregation Effects

Platelets adhere to the endothelial monolayer and release a variety of inflammatory mediators, which can promote activation of the endothelial cells and recruitment of circulating blood cells. Thus, platelets form an inflammatory spot to constitute an early trigger for atherosclerotic lesions. Zuo et al. reported that Rb2 and Rd2 significantly inhibited ADP-induced human platelet aggregation through P2Y_12_-mediated cAMP/PKA and PI3K/Akt/Erk1/2 signaling pathways [89]. Rg3 inhibited platelet aggregation, [Ca^2+^]_*i*_ mobilization, and ATP release of collagen-induced platelets by the PI3K/Akt pathway [90]. Ro showed the calcium antagonist effect, which inhibited platelet aggregation and [Ca^2+^]_*i*_ mobilization of human platelets induced by thrombin [91]. The antiplatelet aggregation and antithrombosis effects of PTS including Rg1, R1, and Re were stronger than those of total saponins from Panax notoginseng in the middle cerebral artery occlusion model rat by regulating the glycoprotein Ib-*α* to reduce von Willebrand factor-mediated platelet adhesion [92]. PTS also inhibited platelet aggregation induced by collagen, thrombin, and ADP via inhibiting [Ca^2+^]_*i*_ mobilization and ERK2/p38 activation [93]. Rg1 inhibited platelet aggregation induced by various agonists including thrombin, ADP, collagen, and U46619 through affecting *α*IIb*β*3-mediated outside-in signaling, and Rg1 also attenuated the arterial thrombus formation by inhibiting the activation and adhesion of platelets [94]. Gao et al. compared the effect of some monomer saponins on ADP-induced platelet aggregation; they found that Rg2, Rh2, Rg3, Rg1, Re, Rd, and nFc obviously inhibited platelet aggregation; however, nFt1, PPD, nFe, and nR1 could promote ADP-induced rat platelet aggregation [95]. Zhang et al. verified the abovementioned results of PPD and 20(S)-panaxadiol (PD); they could induce the platelet aggregation and activation of human and rat platelets with 1 mM Ca^2+^, but the process required the participation of calcium ions. PD and PPD only slightly increased the platelet activation and cannot directly induce the platelet aggregation in vitro [96, 97]. To summarize, we thought that ginsenosides and aglycone exhibited opposite activity on the platelet.

#### 5.4.2. Effects on the Abnormal Vascular Remodeling

Abnormal vascular remodeling is a vital pathological event in cardiovascular disease. Ginsenosides obviously improved vasodilation dysfunction and abnormal vascular remodeling in cardiovascular diseases by affecting calcium ion channels [98]. Rb1 and Rd inhibited mitochondrial swelling of cardiomyocytes induced by Ca^2+^, which protected the heart against I/R and H/R injury by inhibiting mitochondrial permeability transition pore (mPTP) opening and restoring subsequent loss of mitochondrial membrane potential [99]. Rd was a voltage-independent Ca^2+^ entry blocker that inhibited basilar hypertrophic remodeling in stroke-prone renovascular hypertensive rats [100]. PPD caused vasodilation of endothelium-intact aortic rings by activating the Ca^2+^-activated K^+^ channel [101]. Rg1 significantly inhibited [Ca^2+^]_*i*_ overload, loss of mitochondrial membrane potential, and ROS production of astrocytes induced by H_2_O_2_. Angiogenesis plays an important role in ischemic cardiovascular disease, which recuperates the blood flow in the ischemic boundary and improves endogenous neurogenesis and neurological function. Rg1, a potent proangiogenic agent, induced the angiogenesis by targeting RUNX2 and increasing the vascular endothelial growth factor receptor-2 (VEGFR-2) in ischemic injury [102, 103], which reduced the myocardial fibrosis and left ventricular hypertrophy by increasing the expression of HIF-1 and VEGF [104]. R1 significantly restored cerebral blood flow and mitochondrial energy metabolism and promoted angiogenesis after ischemic stroke by regulating the NAMPT-NAD^+^-SIRT1 cascade and Notch/VEGFR-2 signaling pathways [105]. Re showed the antiangiopathy effects by activating the p38 MAPK, ERK1/2, and JNK signaling in the T1DM and T2DM rats [106, 107]. PDS and PTS showed different therapeutic effects on cardiovascular diseases. Some ginsenosides of PTS, such as R1, Rg1, and Re showed proangiogenic action by regulating the VEGF-KDR/Flk-1 and PI3K-Akt-eNOS signaling pathways in ischemic cardiovascular disease [107, 108]. However, some ginsenosides of PDS, such as Rg3, Rh2, and Rd, inhibited the proliferation, migration, and vessel-like tube formation of HUVECs induced by VEGF via inhibiting VEGFR2-Gab1 signaling and Akt/mTOR/P70S6 kinase signaling [109, 110]. Migration of VSMC from their primary site and proliferate, which will lead to neointimal formation with subsequent vascular remodeling via a series of cellular cascades. Rg3 exhibited stronger antiproliferation and antimigration effects of VSMC in diabetic atherosclerosis [111]. Rk1 increases the phosphorylation of myosin light chain and cortactin to reduce the vessel leakiness of the retina in DM mice [112]. PTS mainly influences vascular remodeling of the heart and brain, and the antiangiogenesis of PDS is more conducive to treat diabetes and cancer.

### 5.5. Effect on the Nervous System

#### 5.5.1. Effect on the Neuroprotection

PDS and PTS not only boost memory and learning but also treat neurological diseases. PDS and PTS may play a central nervous system modulatory function by interacting with hormone receptors. Rb1 had neuroprotective effects by inhibiting GSK-3*β*-mediated C/EBP homologous protein signaling, thereby reducing neuronal apoptosis induced by endoplasmic reticulum stress [113]. Rg3 attenuated Hcy-induced neurotoxicity and protected the vascular endothelial cells via *N*-methyl-D-aspartate receptor (NMDAR) activation and the estrogen receptor (ER), respectively [114]. Rg1 could exert an estrogenic effect by activating extracellular regulated protein kinases (ERK) and Akt signaling, and finally improve memory [115]. Rg1 also significantly improved the neurological deficit, attenuated the damage of the blood-brain barrier by downregulating the expression of aquaporin 4, and reduced the neurological damage induced by cerebral ischemia/reperfusion injury in rats [116]. Rb1 improved neurobehavioral function and neuroprotection after subarachnoid hemorrhage-induced brain injury [117] and also significantly promoted neurite growth in hippocampal neurons and protected neurons from A*β*-induced neurotoxicity [118]. Rd and Re exerted neuroprotective effects by reducing cell loss and degeneration and protecting the length and number of neurites in tyrosine hydroxylase (TH+) cells [119]. Rd could improve learning and memory ability and exhibit neuroprotective effects in model mice, probably through inhibiting the transcription activity of NF-*κ*B, reducing proinflammatory cytokines, and generating protective factors [120]. Rb1 and Rg1 improved the cognitive impairment induced by simulated microgravity in rats; the protective effects of Rb1 are superior to Rg1 in some parameters, such as escape latency, SOD, MDA, GSH-x, cleaved-Cas3 expression of prefrontal cortices [121].

#### 5.5.2. Effect on the Alzheimer's Disease

The predominant characteristics of Alzheimer's disease (AD) are progressive impairments in cognition and behavior, which can cause memory loss and dementia. The concentration of acetylcholine (Ach) and the activity of cholinergic neurons were reduced in the brains of AD patients. An increase in Ach via inhibiting acetylcholinesterase (AChE) and butyrylcholinesterase (BChE) could alleviate AD in the initial and moderate stages [122]. Razgonova et al. reported that the inhibition ability of ginsenosides on the activity of AChE was Re > Rg3 > Rg1 > Rb1 > Rb2 > Rc, Rg3 > Rg1 > Rb2 > Rb1 for BChE [123]. PDS and PTS have a remarkably inhibitory effect against AChE and BChE. However, the inhibition effect of PTS was stronger than that of PDS on the AChE and BChE. Wang et al. also demonstrated the opinion that Rg1 strongly inhibited AChE activity compared with Rb1 [124]. Rg1 increased the ratio of Bcl-2 to Bax and the expression of neuronal markers MAP2 and NeuN and decreased the expression of caspase-3, GSK-3*β*, and *β*-catenin, which could alleviate oxidative stress damage, ameliorate neuroinflammation, and protect neurons by regulating the Wnt/GSK-3*β*/*β*-catenin signaling pathway [125]. Rg1 also could repair dendrite and axon and inhibit microglia- and astrocyte-related inflammations in the early- and middle-phases Alzheimer's disease [126]. Kim et al. reported that Re and Rd selectively upregulated the expression of choline acetyltransferase (ChAT) and vesicular acetylcholine transporter (VAChT), which promoted neuron-like cell differentiation and alleviated the symptoms and progression of AD through cell cycle in neuronal differentiation and the nerve growth factor (NGF)-TrkA signaling pathway; moreover, the activity of Rd was superior to that of Re [127]. A*β* peptides are the major indicators of AD. A*β* deposition is generated by cleavages of *β*-amyloid precursor protein (APP) in AD patients' brains. The inhibition ability of ginsenosides on the activity of *β*-site amyloid precursor protein cleaving enzyme 1 (BACE1) was Rc > Rb2 > Rb1 > Rg1 [123], and PDS obviously inhibited the activity of BACE1. Rb1 could relieve cognitive deficits by decreasing expressions of IL-1*β*, A*β*, and glial fibrillary acidic protein (GFAP) and alter the amyloidogenic process of APP into the nonamyloidogenic process in AD rats by exerting anti-inflammatory [128]. Disturbed energy metabolism adversely affects AD pathology. CK was used as a prophylactic or therapeutic agent for AD by promoting the activity of glucose transporters (GLUTs) to increase ATP levels and inhibiting neuronal damage by A*β* peptides through activation of energy metabolism signaling pathways and Nrf2/Keap1 signaling pathway [129, 130]. Both PDS and PTS improve Alzheimer's disease; however, they act through different mechanisms.

### 5.6. Antidiabetes

Diabetes is one of the major global health problems and there is an especially high rate of incidence in the elderly population. It has been shown that ginsenosides, especially PDS and PTS possess antihyperglycemic activity *in vitro* and *in vivo*. Deng et al. reported that PDS and PTS treated the high-fat diet/streptozocin-induced type 2 diabetes mellitus (T2DM) mice by inhibiting expression of hepatic metabolism genes (peroxisome proliferator-activated receptor gamma coactivator 1-alpha, phosphoenolpyruvate carboxykinase, and glucose-6-phosphatase) and promoting the expression of lipid metabolism genes (microsomal triglyceride transfer protein). PDS exhibited a better effect on the improvement of T2DM than PTS in most indicators, such as body weight, blood glucose, ITT, serum insulin content, C peptide, TNF-*α*, and IL-6 [131]. Rb1 reduced 11*β*-hydroxysteroid type I dehydrogenase (11*β*-HSD1) levels in liver and adipose tissue, attenuated insulin sensitivity and elevated blood glucose in T2DM mice [132]. Rg1 improved blood glucose levels and insulin resistance index, as well as lipid distribution and liver function of T2DM mice [133]. 20(R)-Rg3 treatment not only attenuated fasting blood glucose (FBG) levels and advanced glycation end products (AGEs) levels but also improved insulin (INS) levels, blood lipids, oxidative stress, and renal function by regulating MAPKs and NF-*κ*B signal pathways in diabetic nephropathy mice [134]. Rg3 enhanced islet cell function and attenuated islet cell apoptosis in mice, and insulin secretion was 2.3-fold higher in the 4 *μ*M Rg3-treated islet cells compared with the untreated reference islet cells [135]. Rb1 exerted protective effects on diabetes and diabetic complications by regulating mitochondrial energy metabolism, improving insulin resistance, and alleviating the occurrence of complications [136]. Rb1 also regulated the glycolipid metabolism and improved the insulin and leptin sensitivities to exert the antiobesity and antihyperglycemic. Re reversed the insulin resistance and glucose transporter 4 translocation in high-fat diet-induced rats; however, the effect of Re on insulin-stimulated glucose transport was specific, which did not change the skeletal muscle glucose transport induced by contraction and hypoxia [137]. Chen et al. thought that the antidiabetic effects of ginsenosides are positive for T2DM but have no significant improvement in prediabetes or healthy adults [138].

## 6. Conclusions and Future Perspectives

In conclusion, PDS and PTS are important active ingredients of ginseng with strong pharmacological activities, including antioxidant, antifatigue, antiaging, immunomodulation, antitumor, cardiovascular, neuroprotection, and antidiabetic. In recent years, the chemical composition, pharmacokinetics, hydrolytic metabolism, and pharmacological activities of PDS and PTS have been extensively studied, and some important progresses have been made. Due to the difference in chemical structure between PDS and PTS, the absorption, distribution, elimination metabolism, and bioavailability of PDS and PTS are obviously different *in vivo* and *in vitro*. The absorption and bioavailability of PDS is higher than those of PTS *in vivo*; and the elimination rate of PTS is faster than that of PDS. Meanwhile, the absorption and bioavailability of ginsenosides were increased with a reduction in the number of glycosides. The systemic exposure of PDS was significantly greater than that of PTS after oral administration of ginsenosides, such PDS needs widespread attention in clinical for optimizing dosage regimens and predicting outcomes in patients receiving ginseng-based therapy. We summarized the absorption, metabolism, biotransformation, and pharmacological effects of PDS and PTS, and pharmacokinetic parameters of PDS and PTS are closely related to drug activity. It is hoped that the information gathered will facilitate further discussion and research on PDS and PTS.

## Figures and Tables

**Table 1:** Structure characteristics of PDS and PTS.

Type	No.	Name	R1	R2	References
PPD-type ginsenosides	1	20S-ginsenoside Ra1	-glc^3^(2-1)glc	-glc (6-1)ara(p)^2^(4-1)xyl^4^	[9]
	2	20S-ginsenoside Ra2	-glc(2-1)glc	-glc(6-1) ara(f)^1^(2-1)xyl	[9]
	3	20S-ginsenoside Ra3	-glc(2-1)glc	-glc(6-1)glc(3-1)xyl	[9]
	4	20S-ginsenoside Ra4	-glc(2-1)glc(6)Bu^8^	glc(6-1)ara(p)(4-1)xyl	[10]
	5	20S-ginsenoside Ra5	-glc(2-1)glc(6)Ac^7^	-glc(6-1)ara(p)(4-1)xyl	[10]
	6	20S-ginsenoside Ra6	-glc(2-1)glc(6)Bu	glc(6-1)glc	[10]
	7	20S-ginsenoside Ra7	-glc(2-1)glc(6)Bu	-glc(6-1)ara(p)	[10]
	8	20S-ginsenoside Ra8	-glc(2-1)glc(4)Bu	-glc(6-1)ara(f)	[10]
	9	20S-insenoside Ra9	-glc(2-1)glc(6)Bu	-glc(6-1)ara(f)	[10]
	10	20S-ginsenoside Rb1	-glc(2-1)glc	-glc(6-1)glc	[4]
	11	20S-ginsenoside Rb2	-glc(2-1)glc	-glc(6-1)ara(p)	[11]
	12	20S-ginsenoside Rb3	-glc(2-1)glc	-glc(6-1)xyl	[11]
	13	20S-ginsenoside Rc	-glc(2-1)glc	-glc (6-1)ara(f)	[4]
	14	20S-ginsenoside Rd	-glc(2-1)glc	-glc	[4]
	15	20S-ginsenoside Rg3	-glc(2-1)glc	-H	[11]
	16	20R-ginsenoside Rg3	-glc(2-1)glc	-H	[11]
	17	20R-ginsenoside Rh2	-glc	-H	[4]
	18	20S-ginsenoside Rh2	-glc	-H	[4]
	19	20S-ginsenoside Rs1	-glc(2-1)glc(6)Ac	-glc(6-1)ara(p)	[9]
	20	20S-ginsenoside Rs2	-glc(2-1)glc(6)Ac	-glc(6-1)ara(f)	[9]
	21	20S-ginsenoside Rs3	-glc(2-1)glc(6)Ac	-glc(6-1)ara(f)	[9]
	22	Malonyl-20S-ginsenoside Ra3	-glc(2-1)glc(6)mal^6^	-glc(6-1)glc(3-1)xyl	[9]
	23	Malonyl-20S-ginsenoside Rb1	-glc(2-1)glc(6)mal	-glc(6-1)glc	[12]
	24	Malonyl-20S-ginsenoside Rb2	-glc(2-1)glc(6)mal	-glc(6-1)ara(p)	[12]
	25	Malonyl-20S-ginsenoside Rc	-glc(2-1)glc(6)mal	-glc(6-1)ara(f)	[12]
	26	Malonyl-20S-ginsenoside Rd	-glc(2-1)glc(6)mal	-glc	[12]
	27	Malonyl-20S-notoginsenoside R4	-glc(2-1)glc(6)mal	-glc(6-1)glc(6-1)xyl	[9]
	28	20S-gypenoside XVII	-glc	-glc (6-1)glc	[9]
	29	20S-notoginsenoside-Fe	-glc	-glc (6-1)ara(f)	[9]
	30	20S-notoginsenoside R4	-glc(2-1)glc	-glc (6-1)glc (6-1)xyl	[9]
	31	20S-pseudoginsenoside RC1	-glc(2-1)glc(6)Ac	-glc	[9]
	32	20S-quinquenoside R1	-glc (6-1)glc	-glc (2-1)glc (6)Ac	[9]
	33	20S-vinaginsenoside R16	-glc (2-1)xyl	-glc	[9]
	34	20S-quinquenoside I	-glc (2,1)glc-6-butenyl	-glc	[13]
	35	20S-quinquenoside II	-glc (2,1) glc-6-octenyl	-glc(6,1)glc	[13]
	36	20S-quinquenoside V	-glc (2,1)glc	-glc(6,1)glc(4,1)[alpha-D]glc	[13]
	37	20S-quinquenoside R1	-glc (2,1) glc-6-Ac	-glc (6,1) glc	[9]
	38	20S-quinquenoside L10	-glc	-glc (6,1)Ara(p)	[14]
	39	20S-quinquenoside L14	-glc (2,1) glc	-Ara(p)	[14]
	40	20S-ginsenoside F2	-glc	-glc	[4]
	41	20S-pseudoginsenoside F8	-glc (2,1)glc-6-Ac	-glc (6,1)Ara(p)	[9]
	42	20S-notoginsenoside S	-glc (6,1)ara(f) (5,1)Xyl	-glc (2,1)glc (2,1)Xyl	[9]
	43	20S-vinaginsenosideR18	-glc (2,1)glc	-Xyl	[9]
	44	20S-gypenosideXIII	-H	-glc (6,1)Xyl	[9]
	45	20S-gypenosideXVII	-glc	-glc (6,1)glc	[13]
	46	20S-gypenosideXV	-glc (2,1)Xyl	-glc (6,1)Xyl	[9]
	47	20S-gypenosideIX	-glc	-glc (6,1)Xyl	[9]
	48	20S-notoginsenosideK	-glc (6,1)glc	-glc	[13]
	49	20S-notoginsenoside D	-glc (2,1)glc (2,1)Xyl	-glc(6,1)glc (6,1)Xyl	[13]
	50	20S-notoginsenoside O	-glc	-glc (6,1)Xyl (3,1)Xyl	[15]
	51	20S-notoginsenoside P	-glc	-glc (6,1)Xyl (4,1)Xyl	[15]
	52	20S-notoginsenoside Q	-glc (2,1)glc (2,1)Xyl	-glc (6,1)Xyl (4,1)Xyl	[15]
	53	PPD	-H	-H	[11]

PPT-type ginsenosides	1	20S-ginsenoside Re	-glc (2-1)rha^5^	-glc	[4]
	2	20S-ginsenoside Re1	-glc	-glc (3-1)glc	[9]
	3	20S-ginsenoside Re2	-glc (3-1)glc	-glc	[9]
	4	20S-ginsenoside Re3	-glc	-glc (4-1)glc	[9]
	5	20S-ginsenoside Re4	-glc	-glc (6-1)ara(f)	[9]
	6	20S-ginsenoside Re6	-glc	-glc (6)Bu	[9]
	7	20S-ginsenoside Rf	-glc (2-1)glc	-H	[4]
	8	20S-ginsenoside Rg1	-glc	-glc	[4]
	9	20S-ginsenoside Rg2	-glc (2-1)rha	-H	[4]
	10	20R-ginsenoside Rg2	-glc (2-1)rha	-H	[4]
	11	20-gluco-20S-ginsenoside Rf	-glc (2-1)glc	-glc	[9]
	12	20S-ginsenoside Rh1	-glc	-H	[12]
	13	20R-ginsenoside Rh1	-glc	-H	[12]
	14	20S-koryoginsenoside R1	-glc (6-1)Bu	-glc	[9]
	15	20S-notoginsenoside N	-glc (4-1)glc	-glc	[9]
	16	20S-notoginsenoside R1	-glc (2-1)xyl	-glc	[12]
	17	20S-notoginsenoside R2	-glc (2-1)xyl	-H	[12]
	18	20S-yesanchinoside D	-glc (6)Ac	-glc	[9]
	19	20S-chikusetsusaponin L10	-H	-glc	[9]
	20	20S-chikusetsusaponin FK1	-glc (2-1)rha	-glc	[9]
	21	PPT	-H	-H	[12]

Note. ara(f)^1^: *α*-L-arabinofuranosyl; ara(p)^2^: *α*-L-arabinopyranosyl; glc^3^: *β*-D-glucopyranosyl; xyl^4^: *β*-D-xylopyranosyl; rha^5^: *α*-L-rhamnopyranosyl; mal^6^: malonyl; Ac^7^: acetyl; Bu^8^: trans-but-2-enoyl.

## Data Availability

All data generated in this study are included within the article or are available from the corresponding author upon reasonable request.

## Abbreviations

PDS: Protopanaxadiol-type saponins
PTS: Protopanaxatriol-type saponins
glc: Glucose
rha: Rhamnose
xyl: Xylose
ara: Arabinose
PPD: Protopanaxadiol
PPT: Protopanaxatriol
MRT: Mean retention time
OS: Oxidative stress
ROS: Reactive oxygen species
SOD: Superoxide dismutase
GPx: Glutathione peroxidases
GR: Glutathione reductase
MDA: Malondialdehyde
RPE: Retinal pigment epithelial
GSH-Px: Glutathione peroxidase
GSH: Glutathione
NO: Nitric oxide
TNF-*α*: Tumor necrosis factor-*α*
IL-6: Interleukin 6
IL-1*β*: Interleukin1*β*
LDH: Lactic dehydrogenase
FST: Forced swimming test
LPO: Lipid peroxide
TC: Total cholesterol
TG: Triglyceride
EGFR: Epidermal growth factor receptor
CKMM: Creatine kinase muscle type
SIRT1: Sirtuin 1
CP: Cyclophosphamide
Th1: Th1 cell
Th2: Th2 cell
IL-2: Interleukin-2
IFN-*γ*: Interferon-*γ*
IgA: Immunoglobulin A
IgM: Immunoglobulin M
IgG: Immunoglobulin G
CIA: Collagen-induced arthritis
TLR4: Toll-like receptors 4
DC: Dendritic cells
NRF2: Nuclear factor erythroid2-related factor 2
ERK: Extracellular regulated protein kinases
MDA-MB231 cells: Human breast cell line MDA-MB-231
MCF-7: Michigan cancer foundation -7
THP-1: Human myeloid leukemia mononuclear cells
HepG2: Human hepatocellular carcinoma cell
ER: Endoplasmic reticulum
HCC: Hepatocellular carcinoma
TRAIL: TNF-related apoptosis-inducting ligand
JNK: JUN *N*-terminal kinases
EMT: Epithelial-mesenchymal transition
MMP-9: Matrix metallopeptidase 9
AS: Atherosclerosis
ECs: Endothelial cells
VSMC: Vascular smooth muscle cell
ADP: Adenosine diphosphate
ATP: Adenosine triphosphate
PARP: Poly ADP-ribose polymerase
RUNX2: Runt-related transcription factor 2
VEGFR-2: Vascular endothelial growth factor receptor-2
HIF-1: Hypoxia inducible factor-1
VEGF: Vascular endothelial growth factor
HUVECs: Human umbilical vein endothelial cells
VSMC: Vascular smooth muscle cells
C/EBP: CCAAT/enhancer-binding protein
NMDAR: *N*-methyl-D-aspartate receptor
AD: Alzheimer's disease
APP: Amyloid precursor protein
GFAP: Glial fibrillary acidic protein
A*β*1-42: Amyloid-*β*1-42
BDNF: Brain-derived neurotrophic factor
CREB: cAMP response element binding
CCl4: Carbon tetrachloride
TH+: Tyrosine hydroxylase
SAH: Subarachnoid hemorrhage
Ach: Acetylcholine
NGF: Nerve growth factor
CK: Compound K
GLUTs: Glucose transporters
A*β*: Amyloid *β*
PD: Parkinson's disease
MPTP: 1-methyl-4-phenyl-1,2,3,6-tetrahydropyridine
GLT-1: Glutamate transporter-1
11*β*-HSD1: 11*β*-hydroxysteroid type I dehydrogenase
FBG: Fasting blood glucose
AGEs: Advanced glycation end products
INS: Improved insulin
GLUT4: Glucose transporter 4.
